# Dental Robotics: A Disruptive Technology

**DOI:** 10.3390/s21103308

**Published:** 2021-05-11

**Authors:** Paras Ahmad, Mohammad Khursheed Alam, Ali Aldajani, Abdulmajeed Alahmari, Amal Alanazi, Martin Stoddart, Mohammed G. Sghaireen

**Affiliations:** 1 Department of Oral Medicine, Dental Section, Akhtar Saeed Medical and Dental College, Lahore 53720, Pakistan; docparas2017@gmail.com; 2 Department of Orthodontics, College of Dentistry, Jouf University, Sakaka 72345, Saudi Arabia; 3 Prosthodontic Dentistry Department, College of Dentistry, Jouf University, Sakaka 72345, Saudi Arabia; dr.alaldajani@jodent.org (A.A.); dr.a.alahmari@jodent.org (A.A.); amal-6677@hotmail.com (A.A.); dr.mohammed.sghaireen@jodent.org (M.G.S.); 4 AO Research Institute Davos, 7270 Davos Platz, Switzerland; martin.stoddart@aofoundation.org

**Keywords:** dentistry, robots, robotics

## Abstract

Robotics is a disruptive technology that will change diagnostics and treatment protocols in dental medicine. Robots can perform repeated workflows for an indefinite length of time while enhancing the overall quality and quantity of patient care. Early robots required a human operator, but robotic systems have advanced significantly over the past decade, and the latest medical robots can perform patient intervention or remote monitoring autonomously. However, little research data on the therapeutic reliability and precision of autonomous robots are available. The present paper reviews the promise and practice of robots in dentistry by evaluating published work on commercial robot systems in dental implantology, oral and maxillofacial surgery, prosthetic and restorative dentistry, endodontics, orthodontics, oral radiology as well as dental education. In conclusion, this review critically addresses the current limitations of dental robotics and anticipates the potential future impact on oral healthcare and the dental profession.

## 1. Introduction

Robots are machines that perform automatic manual tasks programmed by a computer. Technological advancements in robotics and artificial intelligence have automated more and more tasks, in particular laborious and tedious jobs. Robots can clean floors, trim grass, greatly assist in industrial production, and are becoming an integral part of our daily and professional lives [1,2]. Robots deliver consistently high-performance, economic relevancy and increasingly serve as smart assistants and co-workers in many public areas [3,4].

There is a high demand for improving healthcare efficiency and quantity while standardizing current methods. Dentistry offers multiple opportunities for robotic automation and assistive technology to enhance the quality of dental care. Robots may relieve human resources for more important tasks, such as interacting with patients or other functionalities requiring high cognitive skills [1]. Although dental robots may help improve treatment precision and outcome, robotic transformation is still facing diverse types of challenges [5]. 

The use of robots in dental clinics, especially in the tasks of dental assistants, may constitute one of the most important arguments for robotic dentistry [6]. A study at Oxford University found the tasks of dental hygienists and dental assistants were more likely to be computerized than the tasks of dentists [7]. Jenkins perceived in 1967 as a robotic dental secretary, and several robot applications in dentistry have since become a reality [8]. Dental professionals may experience physical and mental exhaustion after hours of demanding procedures in ergonomically challenging positions, potentially leading to mistakes in the oral examination, disease diagnosis, and treatment planning. There is also a risk of general carelessness surrounding daily routine works, such as cleaning of instruments and surfaces in the dental clinic. Digital medicine/dentistry compatible with robotics can help to minimize errors and enhance the overall quality and quantity of patient care [6,7,8].

Apart from serving as dental assistants, robots in conjunction with 3D navigation can be used for invasive dental procedures, including tooth preparation and autonomous dental implant placement [9,10,11]. Robotic systems can also play a role in education. Training dental students with the aid of full-body robotics, haptic interface technology, and advanced simulation can teach basic learning needs before interaction with real patients [12,13,14,15,16].

## 2. History of Robotics

The pioneering work conducted at the National Aeronautics and Space Administration (NASA) marks the origin of robot-assisted surgery. In the mid-1980s, a remotely controlled robotic system was developed by NASA for surgically operating soldiers on the battlefield as well as astronauts in space. In 2000, the USA Food and Drug Administration (FDA) approved the first robotic system for performing laparoscopic surgery in a doctor-robot setup. In 2001, the validation of the doctor-robot concept was performed via a transcontinental live robotic cholecystectomy. It was the first instance that a team of surgeons operated on a patient elsewhere (telepresence) [17]. Since then, robots have been employed in surgical specialties such as general surgery, gynecology, and urology. Robot-assisted surgery has also prompted progress in minimally invasive surgery by providing equipment with high accuracy and freedom of movement, elimination of the negative effect of instrumental and hand tremors, and real-time stereoscopic vision of the surgical area. Robots offer surgeons easier operational access and flexible working settings [18]. The medical robotic industry has lately been shifting its focus to autonomous robotic technology, this is robots capable of performing a procedure themselves without the constant control or active monitoring by a natural person. Also, remote-control injectable medical microrobots have been envisioned for the delivery of cytotoxic agents to cancer cells.

## 3. Robots in Dentistry

Robots are not used as extensively in dentistry as in medicine. Dentistry employs a few manual robotic systems that are managed manually via the control interface of the computer. Manual robots can provide safer and more accurate drilling than traditional dentistry [19,20]. Progress is being made towards autonomous robots in implant dentistry [21], but the few promising robotic systems are not yet available to dentists [22,23]. High acquisition cost and innate intricacy of the robotic hardware and methods must be overcome before robots will become commonplace in dental practice.

### 3.1. Dental Implantology

The outcome of dental implant treatment is heavily dependent on the precision of implant placement. To reduce errors of implant positioning, dentists use surgical template guidance and navigation systems. However, the site of a missing tooth and limitations in mouth opening may create awkward working positions, potentially causing operator fatigue and human errors. Robot-assisted implant surgery allows for increased flexibility, stability, and accuracy of implant placement [18].

The formation of computer-assisted dental implantology established on a merging of prosthodontics and dental implantology the idea of CT-scan analysis and prosthetic-driven implant dentistry has been investigated. Robotic implant systems usually require surgical tracking in real-time for accurate implant placement [18].

In 2002, Boesecke et al. [24] presented the first robot-guided placement of dental implants. The robot system, having a working region scope of 70 cm, executed the implant drilling guide to help the surgeon during implant osteotomy, where 48 dental implants were placed within 1–2 mm of the apical border [24].

In 2012, an autonomous robotic system that has 6 degrees of freedom (DOF) used a volume-decomposition-based system to place a root-shaped dental implant [25]. Subsequently, a 3-DOF robotic system with a stereo camera was developed that could detect and modulate the dental handpiece to ensure implant placement according to the preoperative protocol [26]. The planned surgical procedure was applied automatically by the computer to ensure the correct cutting site and properly applied force [26].

In 2017, YOMI^TM^ (Neocis, Miami, FL, USA) became the world’s first computerized navigation robotic system approved by the FDA to augment the clinical accuracy of dental implant surgery [27]. YOMI provided physical guidance of the drill’s depth, orientation, and position, thereby avoiding custom fabrication of surgical guide and hand deviation of the operator. The navigation system delivers high predictability and precision while preparing dental implant osteotomy employing vibrational feedback. However, the YOMI system is relatively expensive and operates under supervision [27].

In 2017, Zhao introduced the world’s first autonomous implant placement system [28]. Surgical procedures were executable without any intervention by a dentist, and surgical tasks can be modified automatically with a high degree of autonomy [28]. However, validation data are few regarding the feasibility and reliability of the implant positioning, and the robot’s intelligence decisions.

The Fourth Military Medical University Hospital (Xi’an, China) and Beijing University also developed an autonomous dental implant robot in 2017. The robot system aimed at preventing surgical errors and addressing a shortage of highly competent dentists in China [28]. The system included a mechanical robot, DentalNavi software, an implantation foundation, and an image-guided foundation. The robot, the operation foundation, and the coordinates of patients, to calibrate with the image-guided system, used four kinds of teeth defect models as spatial mapping tools. After placement of implants into the dentition defect models, the precision was assessed by comparing postoperative cone-beam computerized tomography with the planned preoperative trajectory. The outcome was excellent with a mean entry deviation of 0.705 mm ± 0.145 mm, a mean apical deviation of 0.998 mm ± 0.232 mm, and a mean axial deviation of 2.077 mm ± 0.455 mm [28].

Another autonomous surgical robotic system studied in vitro insertion of 5-cm long zygomatic implants in edentulous maxillae and recorded an elevated level of precision in the implant placement [29]. Another study found that a 6-axis robotic arm could enhance the precision of the surgery in zygomatic dental implant placement [30]. The authors suggested that force feedback application utilizing a haptic tool might improve the robotic system [30].

Robotic implantology has also been used successfully in complicated implant cases with significantly decreased alveolar bone [25]. Other studies used industrial robots in phantom experiments of robot-assisted placement of dental implants [9,31]. The industrial robots, possessing 6-DOF, showed an insertion variation of 1.42 ± 0.70 mm and a reproducible implant positioning within ±0.02 mm [32].

### 3.2. Oral & Maxillofacial Surgery

Malignant lesions of the oropharynx are not always readily accessible, and conventional treatment must often resort to radiotherapy and/or chemotherapy. Salvage surgery is usually conducted through mandibulotomy with mandibular displacement and lip split. However, robotic oral and maxillofacial surgery has become an attractive possibility, especially in the treatment of oropharyngeal carcinoma [20]. In 2009, the US-FDA approved the da Vinci system for transoral treatment of selected malignant diseases and all non-malignant lesions of the oropharynx, even when located at the base of the larynx and the tongue. Computer-assisted dental implant surgery is used more and more often by clinicians. The system uses the cone-beam computed tomography (CBCT) analysis but also the standardization of bi-dimensional radiographs [33]. Robotic surgery is also carried out in the upper aerodigestive tract reachable via the oral cavity [34,35]. Transoral robotic surgery, having a stereoscopic vision, multi-articulated instruments, and robotic arms, permits intervention of the oropharynx with minimal invasiveness. Robot-assisted surgery can also provide excellent local control in the treatment of low-risk oral squamous cell carcinoma [18].

In addition to the treatment of pathological conditions, transoral robotic surgery has extensively been utilized for the surgical treatment of obstructive sleep apnea. Based on the apnea-hypopnea index, obstructive sleep apnea is categorized into mild, moderate, and severe. When the apnea-hypopnea index is >15, obstructive sleep apnea is linked with high morbidity and mortality. The standard of care for obstructive sleep apnea is continuous positive airway pressure. However, poor patient compliance and intolerance are considerable issues with continuous positive airway pressure treatment. Several surgical options are available for treating obstructive sleep apnea including bariatric surgery and weight control in morbidly obese individuals, maxillomandibular osteotomy, hyoid bone suspension, resection of tongue base employing radiofrequency or CO_2_ laser, uvulopalatopharyngoplasty, and so forth. The results of these surgical modalities are not consistent since, in such patients, multiple levels of airway obstruction are normally found. According to two meta-analyses, a high success rate of 86–100% could be obtained by maxillomandibular advancement. Despite the efficacy of maxillomandibular advancement, several patients might benefit directly from tongue base reduction [36,37].

In 2010, Vicini et al. [38] first proposed transoral robotic surgery for treating obstructive sleep apnea. Their study involved a total of 10 human participants and examined the effectiveness of tongue base reduction with transoral robotic surgery. They reported that robot-assisted surgery resulted in minimal morbidity and was well-tolerated by the patients. Moreover, the apnea-hypopnea index was significantly improved in all 10 participants [38].

The safety and precision in oral and maxillofacial surgery are influenced by human-associated factors including decreased vision, distraction, trembling, or decreased concentration. A report suggested an autonomous robotic system aimed to perform maxillofacial surgery under the supervision of the surgeon [39]. A robot and a navigation module were uninterruptedly incorporated into the system and a drilling test was performed on five 3D printed human jaw models to analyze the position detecting capacity and assess the operational performance. The robot was able to successfully finish the surgery irrespective of the jaw’s position [39].

A 6-DOF robotic arm was proposed as a surgeon aid during orthognathic surgery. Established on 3D information obtained from a CT, positioning must be performed by the surgeon before surgery [40]. Real-time tracking was performed by the robot by registering the patient’s movements during the surgery. A jawbone skull phantom was used to perform preliminary experiments for the orthognathic surgery. According to Woo and colleagues [40], the available software needs to be upgraded and hardware safety improved before automated orthognathic surgery being investigated in human trials.

### 3.3. Prosthetic & Restorative Dentistry

As per the American Dental Association (ADA), around 113 million adult population of America is missing a minimum of 1 tooth, and 19 million do not have any teeth at all [30,41]. After losing natural teeth, the masticatory and vocal functions are severely affected due to alterations in craniofacial morphology. Prosthodontics is required promptly for recovering the craniofacial morphology as well as the normal functioning of edentulous patients and protecting the temporomandibular joint [42]. Robots in prosthetic dentistry could manufacture partial or complete dentures. Rich technique and experience between the skilled dental technician and experienced dentist are incorporated into the software of prosthetic dentistry expert model. Then, robots in prosthetic dentistry realize the fabrication of partial or complete dentures. The research on robots in prosthetic dentistry would be a breakthrough, as well as technical and theoretical innovation. Its successful operation would not only accomplish the quantification of the partial or complete denture, however, also contribute to the progress of prosthetic dentistry [43].

#### 3.3.1. Tooth-Arrangement Robot

For the fabrication of a complete denture, Canadian scientists developed a single-manipulator robotic system utilizing a 6-DOF CRS robot [44,45,46]. A single-manipulator robotic system for tooth arrangement of complete dentures is composed of the following parts: (a) light-sensitive glue; (b) light source device; (c) denture base; (d) control and motion planning; (e) robot modulation software for arranging tooth and a core control system having tooth-arrangement; (f) computer; (g) electromagnetic gripper; and (h) 6-DOF CRS robot [44,47]. A virtual 3D tooth-arrangement software is programmed established on OpenGL and VC++. This virtual 3D tooth-arrangement software carries out the following functions: (a) create or select file related to the medical history of the patient, formulate dental arch curves and a jaw arch by the experience of an expert as per the patient’s jaw arch measures; (b) view 3D virtual teeth on the screen and modify the position of each tooth. The repeatability precision of this robot system is ±0.05 mm, with a maximum line velocity of 4.35 m/s, and a maximum load of 3 kg [43].

The successful system of tooth-arrangement multi-finger hand (TAMFH) is devised based on the MOTOMAN UP6 robot. The workplace analysis, grasping simulation and theory, and structure of TAMFH were investigated. The TAMFH has 3 fingers, with each finger having 3-DOF. The theoretical requirements of tooth-arrangement are met by this multi-finger hand based on the analysis of motion and workplace simulation. However, it was very difficult for the multi-finger hand to grasp and manipulate the artificial teeth precisely as these teeth have a very complex morphology [48,49,50].

A 50-DOF multi-manipulator tooth-arrangement robotic system contains a slipway mechanism, a dental arch generator, and fourteen independent manipulators. Its mechanism of action is easy to regulate, dexterous and simple. The associated studies regarding the kinematic planning and analysis, high accuracy modulation of such robotic system based on software time, and coordinated regulation of dental arch generator are being performed [51,52,53,54,55,56,57]. Utilizing this robotic system, it only takes half an hour to finish the fabrication of the complete denture. The precision of the robotic system is recorded. The repetitive positioning precision for a single multi-manipulator is ±0.07 mm and ±0.10 mm for the entire robotic system [43].

Aiming at the issue of conventional fabrication procedures of the complete denture, an idea of miniature and professional Cartesian type robot system for tooth-arrangement is introduced established on TRIZ theory. ADAMS is used to perform the kinematic analysis and simulation [58,59].

#### 3.3.2. Tooth Preparation

For clinicians, tooth preparation for crown and bridge is routine work, but it is still challenging even after years of clinical experience. The primary challenge is to decrease the tooth sufficiently to make space while applying the least possible harm to healthy tooth substance. For clinicians, the concept of a robotic system utilized for tooth preparation appears sensible and tempting. An in vitro testing of a mechatronic system has been conducted to aid the clinician in tooth drilling. The report demonstrated good outcomes, however, its validation has not been performed so far in the clinical setup. With a mechatronic system, the accuracy of the clinician’s position was 53% more efficient than without it [60].

A tooth preparation robotic system was presented by Yuan and colleagues [61], which consisted of the following hardware parts: (a) a tooth fixture that connects the target tooth with the robotic tool and safeguards the adjoining tooth from laser-cutting; (b) a 6-DOF robotic arm; (c) an efficient low-heat laser appropriate for the preparation of hard tissue; (d) a CAD/CAM software to generate a 3D motion path of the laser and to design the target shape for tooth preparation; and (e) an intraoral 3D scanning machine for obtaining the 3D information of the subject’s teeth fixture, opposing teeth, adjoining teeth, and the target tooth [61].

A system having micro-robots, modulating a picosecond laser device demonstrated a tooth preparation precision that met clinical requirements, had an error of approximately 0.089 ± 0.026 mm [61,62]. A comparison of another tooth preparation robotic system designed for dental veneers having a rotating diamond tool installed on a robot arm was performed with crown preparation carried out by a human clinician [63]. The results demonstrated better outcomes in comparison with the crown preparation performed by the clinician with an average repetition ability of the robotic system of around 40 µm [64,65].

### 3.4. Orthodontics

Malocclusion is a commonly occurring oral condition that influences appearance as well as oral function and health. Moreover, reduced masticatory function leads to several gastrointestinal disorders, such as dyspepsia. The current prevalence rate of malocclusion in China is around 68%. Fixed orthodontic therapy is the most effective and common method to treat malocclusion, and the bending of orthodontic arch-wire (OAW) constitutes the most vital step of this therapeutic technique. Conventional orthodontics is primarily based on manual operation and visual examination. It is not easy to realize the bending of OAW due to the hyper-elasticity of OAW, the ambiguity of manual operation, and the complex morphology of the formed OAW. This has led to a high degree of randomness and several limitations on clinical orthodontics [43].

#### Robotic System for Bending Orthodontic Arch-Wire

SureSmile OAW bending robot consists of a robot installed on to table or base support surface [66]. The OAW or other medical instruments are held by a first gripping tool, and are either integrated into a mobile arm or might be fixed in regards to the base. The second gripping tool is installed to the periphery of the mobile 6-DOF robotic arm with a proximal part also installed to the base as well as to a distal end that could rotate according to the immobile gripping tool about 3 rotational and 3 translational axes. The force sensors, incorporated into the gripping tools, are utilized to identify overbends required to obtain the ultimate morphology of the OAW and might also possess a heating system through which electricity runs via the wire. SureSmile utilizes contemporary 3D computer and imaging techniques to diagnose and plan treatment and employs the robotic system to personalize fixed orthodontic appliances. Detailed treatment planning can be done by simulating the treatment in anticipation. The principal goal of utilizing CAD/CAM is to improve the quality, efficiency, and reproducibility of orthodontic therapy [67,68].

Gilbert introduced LAMDA (lingual arch-wire manufacturing and design aid) for rapid and accurate bending of OAW. Only the motion in the XY plane can be realized by this system, hence it is unable to bend the OAW having closed-loop [69].

Another robot system for bending the OAW is based on MOTOMAN UP6 and consists of the arch-wire bending actuator, computer, and MOTOMAN UP6. The actuator is connected with the periphery of the MOTOMAN robot. The clamping and bending of the arch-wire are done by the arch-wire bending actuator, which is attached with the MOTOMAN robot end [70,71]. The bending characteristics of the arch-wire, the kinematics of the robot, angle optimization, and bending point’s position of the arch-wire are examined and simulated [72,73,74,75,76,77].

The experiment of bending OAW is carried out utilizing a Cartesian type OAW bending robotic system. This robotic system consists of the arch-wire bending system, bending die, supporting structure of the OAW, feed, the rotary, and the base [43,78]. Solidworks software is employed to design the structure of the OAW bending robot. Precision control having a 3rd order pure S acc/dec of OAW bending robot is developed [79,80].

### 3.5. Endodontics

Root canal therapy is a technique that demands high precision and accuracy. Generally, a clinician who specializes in endodontic works utilizing magnification for ensuring sufficient vision of the root canal system. Nelson and colleagues suggested a concept of a robot system to assist while performing root canal therapy. The primary function of the “vending machine”, as proposed by the authors, was to provide the clinician with the required root canal therapy instruments during the procedure [81]. A recently published report suggested the utilization of micro-robots having catalytic-capability to disrupt oral biofilms present inside the root canal and analyzed the robotic system in the laboratory. Moreover, the researchers explained the employment of these robotic systems for other applications including the prevention of peri-implant infection or dental caries [81,82].

### 3.6. Oral Radiology

It is usually perceived that the oral radiologist can penetrate the oral cavity in almost every aspect of the tooth in a minimally invasive manner. Hence, why are robots required? Several benefits of employing robots exist; (a) by the application of robotics, the oral radiologist can be remote to the area where the radiographic procedure is being performed and hence is not predisposed to any radiation exposure; (b) the robotic systems have been developed with many DOF in navigation which makes their dexterity much better as compared to humans. Therefore, in teeth with complex morphology and anatomy, navigation of the radiographic tools would be safer, simpler, and easier [83]. The positioning of the X-ray source and sensor/film was suggested to be performed by a 6-DOF robot arm and no side effect was reported. The outcomes demonstrated that the mechanical alignment technique of the robotic system was superior owing to its remarkable repeatability and accuracy [84]. Burdea et al. [85] devised a robotic system for dental subtraction radiography which employed a 6-DOF position sensor, and a robot arm with an X-ray source was proposed. To identify the effect of sensor and robot errors, an error analysis was carried out. A series of experiments to identify sensor noise and accuracy were explained. According to the findings, there was no adverse effect reported because of the presence of metalwork in the patient’s oral cavity [85]. Another study described the application of a robot having a fully-equipped skull for examining the impact of head motion to the precision of 3D imaging [86].

### 3.7. Dental Hygiene Applications

The removal of plaque by powered or manual toothbrushing is the most efficacious preventive method for controlling oral diseases [87]. Robotic systems might be used to test the effectiveness of toothbrushes along with their abrasion ability towards dental enamel with the highest comparability and repeatability. According to a laboratory study that compared the efficacy of an in vitro robotic brushing with clinical hand brushing, robotic brushing is a comparable technique with clinical hand brushing for the removal of dental plaque and might even replace manual clinical hand brushing [87]. Driesen et al. [88] conducted an in vitro robot study by developing the Braun Oral-B Ultra Plaque Remover (EB5 brush head and D7 handle) which simulates normal clinical toothbrush use. A hybrid new brush head (EB9) having longer bristle tufts developed to enhance interdental penetration, and a higher frequency of bristle motion (63 Hz, 3800 strokes/minute-D9 handle) was associated with a considerably higher removal of artificial plaque [88]. In another study, Gaengler et al. [89] developed a clinically validated robot toothbrushing program for reproducible and fast in vitro testing of tooth cleaning. The authors concluded that robot testing toothbrushing utilizing powered or manual or any brushing method, combined with the computer-assisted planimetrical plaque evaluation is a recommended research tool to develop newer prototypes and comparisons with reference toothbrushes [89]. Similarly, Ernst et al. [90] developed a robot system that simulated 3D brushing movements as a function of time. The in vitro results revealed the capability of the robotic system to exhibit reproducible significant differences in the cleaning efficacies of powered toothbrushes [90].

### 3.8. Dental Education

The concept of a dental training robot was first presented in 1969 [91]. In 2017, the experiment of utilizing a humanoid in dental education was performed. A humanoid (i.e., a complete-body subject simulation robotic system [SIMROID]) was analyzed in a report among dentistry students to examine if a robotic subject (patient) was more realistic compared to the normally utilized dummies for the dental students to get accustomed to real patients [13]. The SIMROID, “Hanako” is 165 cm tall and its skin comes with a vinyl-chloride-based gum pattern with a metal skeleton. “Hanako” is a valuable contribution to dental education as it is emulating a human in its expressions and actions. It can carry out movements of wrist, elbow, tongue, and jaw, shake its head during pain, blink, roll its eyes, and verbally express its discomfort. Moreover, it could also simulate functions for inducing salivary flow and bleeding, and simulate a vomiting reflex using a uvula sensor [92]. Tanzawa and colleagues presented a robot for a medical emergency that aimed to aid dentistry students to get accustomed to emergency conditions [93].

ROBOTUTOR is another dental education robotic system explained in the literature [94]. This robot was an alternative system to a dentist for showing tooth-cleaning procedures (i.e., tooth brushing) to patients. According to a study [94], the patients reported the ROBOTUTOR as the most attractive technique for dental education than other techniques (i.e., audio-video tutorial or dentist). However, the ROBOTUTOR was found to be less efficient as compared to the dentist [94].

According to a study, virtual reality training combined with human instructor verbal feedback and haptic feedback could be the most effective way of learning the fundamental motor skills for dental students [95]. Other reports examined the utilization of haptic devices and virtual reality for the training of oral anesthesia or placement of dental implant [96,97]. In dental education, virtual reality laboratories having haptic devices are gaining much popularity and are becoming an essential part of the regular dental curricula for improving the learning effect and efficiency of the students [95,98,99]. Moreover, a report examined pre-clinical dentistry students’ learning experience utilizing 3D printed human teeth devised with realistic dental pulp cavities and mimicked dental caries [100]. According to the findings of this study, the dental students could develop an entire idea for prosthodontic teeth therapy on simulated 3D printed teeth as these simulated teeth had several characteristics to aid the training of dentistry students [100].

### 3.9. Dental Assistance and Dental Materials

The robotics finds application to standardize simple endoral radiographs to analyze the CBCT and compare the bone volumes to match the bone pre-and post- augmentation procedure [33]. A study examined the probability of active robot assistance during dental procedures by exchanging instruments through a multi-nodal communication model designed for clinicians as consumers. It consists of visual gestures, speech input, touch display input, bilateral physical robot-human communication. In this study, the researchers utilized a state-of-the-art sensitive, collaborative, and safe 7-DOF robotic system and performed a user study to investigate the possibility of varying robot-human communication dental procedures [5].

Robotic mastication or dental wear simulators are suggested to analyze dental implant materials [101,102] or tooth filling materials [103]. A 6-DOF robot drove one of the systems [104,105]. Another report described the testing of dental impression material utilizing a robotic arm [106].

## 4. Is Robotic Dentistry Destructive or Disruptive

Robots are being investigated in several applications ranging from precision treatment to drug delivery [107,108]. Magnetic robotic actuation has caught remarkable attention since it permits tether-free controlled movement together with allowing a wide array of robotic locomotion and mobility techniques [109,110]. Under an adequate environment, magnetic fields harmlessly and easily infiltrate the majority of synthetic and biological materials and can guide particle mobility in the confined area [107]. Although robots have been developed for particular tasks including multimodal locomotion, manipulation of cells and particles, and targeted cargo delivery [111], however, their applications for chemical and physical biofilm disruption remain to be investigated. For instance, Hwang and colleagues [82] designed catalytic antimicrobial robots (CARs) that controllably, effectively, and accurately removed, degraded, and killed biofilms from surfaces. These “kill-grade-and-remove” CAR system might fight persistent biofilm infections and could be used in dentistry.

Change in dentistry is accelerating and will continue to accelerate. For instance, a handful of dentists would utilize alcohol or cocaine toothache drops as a local anesthetic, but today researchers are performing experiments employing nanobots modulated nerve impulse traffic for removing pain sensation. Since the discovery and dental application of radiography in 1895 and 1896, respectively, the exposure time of radiography has decreased from 25 min to a few milliseconds. Flat-plane images are being supplemented by MRI, PET, intra-oral scanning, 3D cone-beam computerized tomography, and other imaging techniques. Before the 1980s, gloves were not mandatory for clinical procedures, the change being principally in reaction to HIV/AIDS [112].

Dental materials and dental restoration approaches have transformed drastically over time. A wax cap, an ancient dental filling, was used to restore a broken tooth over 6500 years ago. Between 25 BC and 50 AD, a book providing a systematic explanation of oral diseases and their interventions was present in the Roman Imperial era. The restoration of the missing Roman tooth was performed using gold wire upholding a substitute tooth with evidence that both ivory and steel were also utilized [112]. Remarkable improvements have been made regarding dental material selection and procedures over time. Branemark’s comprehension of osseointegration induction (1983), Bowen’s resin-based dental composites (1963), Buonocore’s 1955 presentation to the utility of adhesive dentistry are probably the three most revolutionary contemporary developments [113,114,115,116,117,118,119].

Dentistry has survived and thrived over the ages despite all these transformations. Longevity and care of orofacial complex and teeth have improved. Robotic dentistry has altered how clinicians think and perform, and the patient’s experience has improved as well. It has produced a shared workflow for capitalizing on the best skills for varying functions. Unquestionably, the influence of robotic dentistry in no manner is destructive but disruptive.

## 5. Limitations of Robotic Dentistry

When a novel technology is introduced in a new setup, it is subjected to several obstacles of varying nature. One such obstacle is that technological advancements in medical/dental applications are extremely expensive [120]. Furthermore, robotic systems are complex and require expertise for their proper operation and function. Moreover, another vital aspect might be the unknown patient acceptance and compliance among dentists [121]. According to a study, male subjects are more motivated to receive robotic medical therapy as compared to female subjects [121]. Regardless of sex, the motivation to receive such treatment is reduced with the increase in technique invasiveness [121].

The point regarding the input of data is very critical. Presently, in dentistry, we see two main facets—Those that are performed very well via meticulous preoperative planning and implementation, and those carried out very inefficiently by the inexperienced and uninformed personnel resulting in bad (mostly disastrous) results. Hence, the results would only be as good as the personnel incorporating the data into the robotic system.

## 6. Future Recommendations

Throughout the literature, it has been claimed that the application of robots in dentistry improves accuracy, reproducibility, and reliability, however, the quantity of research performed in robotic dentistry is limited due to the lack of accessible systems. Moreover, there is a lack of expertise to program and regulate robotic systems. Therefore, research in this area depends on effective collaboration between dentists and engineers. This might alter shortly since the robotics community investigates innovative communication strategies and programming paradigms.

Unfortunately, the majority of the interdisciplinary research merging dentistry and engineering in robotic dentistry is focused on dental implantology, though the invasive nature of this technology might undermine the acceptance of this application among dentists and patients. Thus, these invasive technologies are less appropriate as forerunners. Hence, research in the domain of assistive robotic dentistry appears to be more potential to support the presentation of this contemporary robotic-enabled era. Moreover, research on dental educational robotics in university setup appears to be a potential propagator to introduce robotic dentistry and removing the hindrance of acceptance of robotic system among future dentists.

All robot applications described in this article from teeth arrangement for complete dentures to dental material testing or tooth preparation have an innate potential to progress dentistry into a new era where digitalization supports the management of our real world. Overall, the level of technological readiness is still less, and more research is required to be performed to produce a worth of robotic dentistry, hence the pace of research in this domain should increase over the next years.

## 7. Conclusions

Dentistry is making its way towards a new world of robot-assisted and data-driven medicine. However, robotic systems have not still been entirely introduced to dental research nor have they achieved cost-effectiveness and technological readiness to be fully incorporated into the dental market. The applications of robots in dentistry, including material testing, orthodontics, prosthodontics, oral surgery, implant dentistry, are promising but the most important limitations for robotic dentistry, besides difficult operating systems and high cost, are fundamental manipulation and sensory abilities of robots and deficiency of learning capabilities. Furthermore, improved intuitiveness of the robotic systems merged with the introduction of the affordable system and extensive educational efforts are key limitations that require to be overcome for the true introduction of robots in dentistry. The dentists should get familiarize with robots including real and digital world robot-human communication skills.

Undoubtedly, manifold possibilities of the future merging of dentistry and robotics exist. The most sooner one is to generate a working system that provides all essential demands including a system that could be used on a large-scale having particular beginning use-cases, intuitive and reliable to utilize manipulation expertise, robot-human communication, human-centered interaction, and human safety [6]. Based on this idea, we highlight the technological progress which allowed the employment of robotics in dentistry. Moreover, we encourage this step by explaining the opportunities that may originate from the merging of robots and dentistry.

## Data Availability

Not Applicable.
